# Inclusion of Cephalexin in COVID-19 Treatment Combinations May Prevent Lung Involvement in Mild Infections: A Case Report with Pharmacological Genomics Perspective

**DOI:** 10.1055/s-0041-1726461

**Published:** 2021-03-22

**Authors:** Amir Khodavirdipour

**Affiliations:** 1 Department of Biology, Faculty of Natural Sciences, University of Tabriz, Tabriz, Iran; 2 Division of Human Genetics, Department of Anatomy, St. John's National Academy of Health, Bangalore, Karnataka, India

**Keywords:** COVID-19 treatment, SARS-CoV-2, cephalexin, omeprazole

## Abstract

Novel coronavirus disease 2019 (COVID-19) is caused by a nonsegmented positive sense RNA, enveloped RNA virus that belongs to the family of β-coronaviridae. This virus shall cause acute respiratory distress syndrome (ARDS) which consequently leads to breathing difficulty and need to admit to intensive care units (ICUs). The current conventional treatment combination in most of the hospitals in Iran includes azithromycin 500 + naproxen 500 + vitamin C 1,000 + Zinc + vitamin D3 1,000. In this case reports ( *n* = 4), we would like to report significant findings in course of COVID-19 treatment reported to our clinic on August 8 and 9, 2020; patients presented as walk in and were advised house isolation and complete bed rest as there were no signs of lung involvement and their overall condition was stable. By the inclusion of cephalexin 500 in treatment combination, patients who received cephalexin 500 for 5 days along with other medicines did not develop any lung involvement and breathing complications. Cephalexin is the gold standard in upper and lower respiratory tract infections and here also shall play a vital role besides other conventional therapies. Azithromycin is a macrodial antibiotic working via the *ABCB1* gene pathway. As of date, there is no clear evidence of pharmacogenomics data in COVID-19 patients. More research needs to be performed in COVID-19 before any sort of pharmacogenomics tests could be advised.

## Introduction

Novel coronavirus disease 2019 (COVID-19) lately named the first pandemic disease of the 21st century. As of January 31, 2021, mortality rate reported above 2,225,000 cases worldwide and numbers are growing rapidly due to the latest dreadful mutations from the United Kingdom to Brazil and South Africa. 1 Disease transmission from human-to-human spread via air droplets by sneezing or coughing from a positive person. 2 To date, prevention is named as the best containment way. People should obey hygienic protocols by following health tips such as no physical contact, using a face mask, washing hands, and keep a safe distance from people in public areas. 3 Diagnosis shall be confirmed by molecular tests (polymerase chain reaction [PCR]) and computed tomography (CT) scans and chest X-rays in case of lung involvement. 4 Reported mortality is different by countries and depends on the way they treat or people following the protocols. People with jobs in public sectors are the most at risk including doctors, nurses, bank tellers, public transport drivers, and others.

## Patients and Methods

Four self-reported young males under age 36 years were included in this study. All of them working in the public sector and equipped daily with face mask, face shield, and gloves, and all samples' exposure levels estimated the same based on the history taken. Due to the Red status issued by the Health Ministry of Iran for more than 75% of Iranian provinces, 5 after the preliminary examination, the samples were taken for the molecular analysis and medication treatment course started and advised for house isolation and complete bed rest as there was no sign of lung involvement. The full history of patients and their advised treatment regimen are available in Table 1 . Interestingly, all of them felt an increase in appetite after the next 48 hours of the first sign of disease. As per World Health Organization (WHO) protocol, as there is no precise treatment option for COVID-19, symptomatic management as per the patient's clinical symptoms and condition is the only choice.

**Table 1:** Timeline of signs and symptoms of all four patients and treatment combinations advised

Timeline	P1–M/31	P2–M/35	P3–M/33	P4–M/36
Day 1	Sore throat	Fever, headache	Sore throat	Sore throat
Day 2	Fatigue, sore throat, runny nose	Headache, sore throat, fatigue	Shivering, high fever, runny nose, PCR+	Fever, anosmia, runny nose
Day 3	PCR+	PCR + , Stomach ache	diarrhea, body ache, anosmia, ageusia, mild fever, CT no lung involvement	Severe sore throat high fever, severe diarrhea, PCR+
Day 4	No cough, no body ache, sore throat, mild fatigue, anosmia	CT no lung involvement, anosmia	Severe fatigue, the coughing started	Stomach ache, fatigue, vertigo, coughing started
Day 5	CT shows no lung involvement	Stomach ache, diarrhea, fatigue	Heavy diarrhea, severe fatigue, body pain, CT 5% lung involvement	–
Day 6	Start to recovery	Mild fatigue	–	CT showing 3% involvement, PCR+
Day 7	–	Start to recovery	–	–
Day 8	–	–	–	Smelling sensation return slowly
Day 9	–	–	–	–
Day 10	–	–	Start to recovery	–
Day 11	–	–	–	Start to recovery
Day 12	–	–	PCR+	–
Day 15	PCR+	PCR+	–	PCR+
Treatment combination	Azithromycin 500 Cephalexin 500 Naproxen 500 Panadol cold flu Zinc Vitamin C 1,000 Vitamin D 1,000	Azithromycin 500 Cephalexin 500 Naproxen 500 Omeprazole 20 Zinc Vitamin C 500 D3 1,000	Azithromycin 500 Naproxen 500, Panadol cold flu Vitamin C 1,000 Vitamin D3 1,000 Zinc	Azithromycin 500 Naproxen 500 Panadol cold flu Vitamin C 1,000 Vitamin D3 1,000 Zinc

Abbreviations: CT, computed tomography; M, male; P, patient; PCR, polymerase chain reaction.

Note: No patients admitted to ICU. All advised to rest at home.

Interestingly all of them felt increase in appetite after next 48 hours of first sign of disease.

## Discussion

**Fig. 1:**
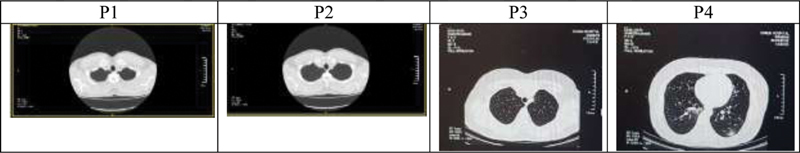
Results of the CT scans illustrated that the patients who were under cephalexin administration along with other conventional therapies showed no lung involvement and breathing complications (patients 1 and 2). Patients 3 and 4 undergone regular symptomatic management regimens. CT, computed tomography; P, patient.

Public sector exposure to COVID-19 is one of the main concerns of transmission after deciding to reopen after March to April quarantine in Iran. At the beginning of COVID-19 in Iran, there was not enough public awareness and protection against the virus. This study showing that not only older people and/or with secondary health problems like cardiovascular diseases (CVD) or kidney diseases can infect but also a young and healthy adult with no previous health issues can also be infected by the virus. All four patients reported positive in their molecular real-time PCR test, while patients 1, 2, and 3 showed no lung involvement in their first CT and patient 4 showed mild lung involvement ( Fig. 1 ). The medication treatment course started on the days 2 and 3 for all four patients. Patients 1 and 2 were advised to take cephalexin 500, one capsule each at every 6 hours for 5 days for cystic pimple and perioral dermatitis, respectively. Despite being PCR positive, none of them developed the breathing complications or lung involvement by COVID-19 and felt overall amelioration on the days 6 and 7 but the other two patients who followed the routine medicinal regimen, felt the same after almost 2 weeks. Jain et al reported that cephalexin can be useful against upper respiratory tract infection. 6 Khodavirdipour et al in a very latest update listed all the treatment combinations suggested against the COVID-19; 7 so, now this breakthrough can be a great help in the fight against COVID-19 for faster recovery time and also keeping lung safe and protect from any sort of complications. Need to mention that patient 2 prescribed to take omeprazole due to his heartburn and stomach ache. Bojkova et al in their recent paper elucidated that omeprazole above the usual clinical dose showing the anti–severe acute respiratory syndrome-coronavirus-2 (SARS-CoV-2) protective capacity. 8 The current study experienced some limitations including a low number of samples. Due to the high seriousness and importance of the matter and triumph in protecting the lungs of the patients, our team felt these findings can be road maps for other colleagues worldwide to save people's lives.

## Pharmacogenomics

Cephalexin is a quick absorber and does it via the intestine through the H ^+^ /depipetide transporter *PEPT1* gene product (solute carrier family 15 member 1 protein) and reabsorbed chiefly from the kidney by *PEPT2* gene product at the basolateral membrane of proximal tubule cells of the renal system. Reportedly, a specific variant of *SLC15A1* showing a remarkable decreased in cephalexin uptake in cells that are transfected. 9

One of the stars of the macrolide antibacterial agent category is azithromycin which has significant anti-inflammatory characteristics. Azithromycin pharmacokinetics elucidate that its activity under the control of P-glycoprotein transported is encoded by the *ABCB1* gene. The variations in *ABCB1* gene such as rs1045642CC/rs2032582GG/rs1045642TT and rs2032582TT. 10

## Conclusion

Despite being one of the oldest and famous antibiotics marketed by 1969, cephalexin is suggested to be administered next to other antibacterial drugs, such as azithromycin, in cases of viral infections such as COVID-19. By considering the above findings, adding cephalexin 500 or even omeprazole to the conventional therapy combination can be worthwhile as the burden of COVID-19 becoming unbearable day by day, as the number of positive cases surges. The situation turning to be exhaustive in terms of human resources, economically and also mentally for health care services, governments, affected individuals, and their families.

As of date, there is no clear evidence of pharmacogenomics data in COVID-19 patients. But there are credible mechanisms which show that the genetic makeup of each person can play critical role in his/her battle against COVID-19. More research needs to be performed in COVID-19 before any sort of pharmacogenomic tests could be advised.
